# Triptolide and l-ascorbate palmitate co-loaded micelles for combination therapy of rheumatoid arthritis and side effect attenuation

**DOI:** 10.1080/10717544.2022.2115162

**Published:** 2022-08-23

**Authors:** Man Li, Guoqiang Wang, Yinyin Yan, Mengyuan Jiang, Zhirong Wang, Zhenqiang Zhang, Xiangxiang Wu, Huahui Zeng

**Affiliations:** a Academy of Chinese Medicine Sciences, Henan University of Chinese Medicine, Zhengzhou, China; b School of Basic Medicine, Henan University of Chinese Medicine, Zhengzhou, China

**Keywords:** Triptolide, vitamin C, toxicity, water solubility, rheumatoid arthritis

## Abstract

Triptolide (TP) has its unique curative effect in the treatment of rheumatoid arthritis (RA), but its application is limited by the poor water solubility and multi-organ toxicity. We herein developed a novel nanoparticle platform composed of L-ascorbate palmitate (VP, vitamin C derivative) that can deliver TP to synergistically treat arthritis and inhibit the occurrence of oxidative stress. The TP-loaded nanoparticles (termed TP-VP NPs) showed the suitable particle size (about 145 nm) and good physical stability. TP-VP NPs effectively down-regulated IL-1β, IL-6 and TNF-α levels to inhibit the erosion of synovitis and bone tissue, and alleviate the swelling and deformation of CIA mice’s feet. Compared to the TP, TP-VP NPs could inhibit effectively the oxidative stress in liver, and alleviate significantly the triptolide-induced toxicity injury in liver, kidney and testicle. The results demonstrated that TP-VP NPs is a promising triptolide delivery system for the treatment of RA, which enhances the water solubility of TP and reduces the toxicity of TP *in vivo*.

## 1. Introduction

Rheumatoid arthritis (RA) is a systemic autoimmune disease of joints, characterized by hyperplasia of synovial joints, joint inflammation, cartilage erosion and bone destruction (Liu et al., 2021). The destruction of cartilage, bone and other adjacent tissues are often irreversible in the later stages of the disease, which will eventually lead to severe joint deformities and even disabilities (Zhao et al., 2021). The global incidence rate of rheumatoid arthritis is about 0.4% to 1.3% (Littlejohn & Monrad, 2018). Currently available anti-rheumatic drugs, including non-steroidal drugs, glucocorticoids, and biological agents, cannot cure RA radically but only relieve its symptoms and progression (Goldbach-Mansky et al., 2009; Weber et al., 2019). In China, herbal medicines have been extensively utilized as alternative anti-rheumatic drugs for decades (Zhao et al., 2019; Li & Zhang, 2020). However, the traditional herbs contain a lot of ingredients which may result in serious side-effects (Wang et al., 2019). Therefore, the functions of every ingredient must be further defined to improve the therapeutic performance of the herbs.

Triptolide (TP) is an epoxidized diterpene lactone compound. It is the main active component in Tripterygium wilfordii polyglycoside which has immunosuppressive, anti-inflammatory, anti-rheumatic and anti-tumor effects. TP has its unique curative effect in the treatment of RA through inhibiting the production of pro-inflammatory cytokines and inflammatory mediators, inhibiting angiogenesis, protecting rheumatoid arthritis cartilage and gene regulation (Liu & Lin, 2006). However, its application is greatly restricted by the poor aqueous solubility, and multi-organ toxicity, which are mainly associated with damaging the liver, kidneys, and reproductive system (Wei et al., 2019; Xie et al., 2020). Thus, it is desirable to explore an effective and safe drug carrier to improve the pharmacological performance of triptolide.

To address these issues, a novel TP nanoparticle was developed for RA treatment. Vitamin C, also known as ascorbic acid, is one of the most effective antioxidants, ascorbic reagents and so on. Some studies have shown that vitamin C plays a significant protective role by maintaining the antioxidant activity of hydroxyl oxidase and reducing reactive oxygen species and tissue oxidative damage. In addition, it can inhibit the triptolide-induced apoptosis in renal tubular epithelial cells by inhibiting the occurrence of oxidative stress (Dennis & Witting, 2017; Xu et al., 2019). Here, triptolide loaded nanoparticles were prepared by using vitamin C derivatives as the carrier, which could reduce some side effects of TP and guarantee the therapeutic effect for RA. Besides, it was confirmed by the toxicity assay *in vivo*, the clinical indexes of the anti-inflammatory effect, and the histological analyses of synovial joints.

## 2. Materials and methods

### 2.1. Materials and animals

Triptolide was purchased from Xi'an Haoxuan Biotechnology Co. Ltd. (Xi'an, China). All other reagents and solvents were purchased from commercial suppliers with analytical grade. Eight-week-old male Kunming mice (40 g) from Beijing Vital River Laboratory Animal Technology were housed under specific-pathogen-free (SPF) condition. The mice were allowed to acclimatize for one week in a light-dark cycle environment at 25 ± 1 °C for 12 h and provided with water and food. All animal experiments were performed in accordance with the guidelines of the Institute’s Animal Care and Use Committee in Henan University of Chinese Medicine.

### 2.2. Preparation of TP-VP NPs

A certain amount of L-ascorbate palmitate (VP), cholesterol, and triptolide were precisely weighed and dissolved in methanol. The organic solvent was removed by rotary evaporation at 40 °C. After forming a uniformly dry film, the residual organic solvent was removed by freeze-drying. Then, some distilled water was added under the stirring, followed by ultrasonic treatment to promote particle dispersion. The nanoparticles (TP-VP NPs) were successively extruded through 200 nm and 100 nm polycarbonate films.

### 2.3. Characterization of TP-VP NPs

#### 2.3.1. Particle size, zeta potential and morphology

Stability of TP-VP NPs was greatly affected by particle size, polydispersity index (PDI) and Zeta potential, which were measured by Nano brook 90Plus PALS at 25 °C. The shape and surface morphology of TP-VP NPs were visualized by Transmission electron microscopy (TEM).

#### 2.3.2. Encapsulation efficiency (EE) and drug loading (DL)

The EE and DL of TP-VP NPs were measured by high-performance liquid chromatography (HPLC) method. The TP-VP NPs reaction solutions were placed in an ultrafiltration centrifuge tube (cut off 3kD), and then centrifuged at 4 °C for 25 min at 4000 r/min. The unencapsulated triptolide (M_free_) in the solution was determined by HPLC. The TP-loaded nanoparticles (100 µL) in the ultrafiltration centrifuge tube was dissolved in 400 µL methanol to destroy the structure of nanoparticles and release TP. The encapsulated triptolide (M_package_) in nanoparticles can be determined by HPLC to calculate the encapsulation efficiency. At the same time, the nanoparticles (100 µL) were freeze-dried and weighed (M_total_). The drug loading and encapsulation efficiency were calculated as the below formulas:

$$\begin{array}{c} \text{EE}\;\left(\%\right)\;=\;\left(M_{\text{package}}\right)/\left(M_{\text{package}}+{\;M}_{\text{free}}\right)\\ \text{DL}\;\left(\%\right)\;=\;\left(M_{\text{package}}\right)/\left(M_{\text{total}}\right) \end{array}$$

where the M_package_ represents the weight of TP loaded in nanoparticles, and M_free_ represents the weight of free TP after centrifugation, and M_total_ is the weight of nanoparticles.

#### 2.3.3. *In vitro* release study

The *in vitro* release kinetics of nanoparticles were investigated using dialysis. The nanoparticle suspensions (1 mL, containing 1.0 mg of TP) and free triptolide solution (1 mL, 1.0 mg/mL) were sealed in a dialysis bag (interception: 1000 Da), respectively. The dialysis bags were immersed into 40 mL of PBS (pH 7.4) containing 0.5% (v/v) of Tween-80 at 37 °C. The dialysate of 2 mL was taken and synchronously restored with the fresh release medium at 0.5, 1, 2, 5, 8, 12, and 24 h, respectively. TP in dialysate was measured by HPLC as mentioned above. The *in vitro* release rate of nanoparticles was calculated. Each assay was repeated in triplicate.

#### 2.3.4. *In vitro* stability of nanoparticles

The stability of nanoparticles was evaluated by particle size and Zeta potential. The nanoparticles were added to PBS or DMEM medium containing 10% serum in shaking incubator at 37 °C, and were oscillated at 100 r/min. The particle size, PDI, and Zeta potential were measured at 2, 4, 8, 12, 24, 36, and 48 h, respectively.

### 2.4. *In vivo* therapeutic efficacy on CIA mice

#### 2.4.1. Preparation of CIA model

The collagen-induced arthritis (CIA) mouse was prepared as follows: Bovine type II collagen (CII) was emulsified with an equal volume of complete Freund’s adjuvant (FA). The CII-containing emulsion (0.2 mL) was injected subcutaneously at the tail root of the mice to induce inflammation. On day 21 after the primary immunization, the emulsion (0.5 mL) containing the equal volume of CII and incomplete Freund’s adjuvant (IFA) was injected into the tail root to enhance immunity. The progression of RA was evaluated by the previously reported standard (Zhang et al., 2020).

#### 2.4.2. Grouping and administration

The CIA mice were randomly divided into 8 groups (*n* = 10), e.g. the model group (CIA mice), the TP groups (treated with TP of 100, 200, 250 µg/kg, respectively), the nanoparticle groups (treated with equal TP of 100, 200, 250 µg/kg, respectively) and the L-ascorbyl palmitate group (at the dose of 50 mg/kg VP). In addition, the model group and the control group (normal mice) were treated with PBS. Each mouse was administered via tail vein injections once every two days. During the treatment, body weight, foot volume, and arthritis index of limb joints were measured every two days. After the treatment, the orbital venous blood was collected for the following experiment, and then mice were sacrificed in accordance to institutional guidelines. The ankle joints, livers, kidneys as well as testis were collected for histological analysis.

#### 2.4.3. Serum inflammatory cytokines detection

When the serum was separated, the levels of TNF-α, IL-6 and IL-1β in the mouse serum were examined by the ELISA kits according to the instructions.

#### 2.4.4. Determination of superoxide dismutase, malondialdehyde and glutathione peroxidase level in the liver

The liver samples were homogenized in saline. The levels of superoxide dismutase (SOD), malondialdehyde (MDA), glutathione peroxidase (GSH-Px) in the liver were determined using the corresponding kits following the manufacturer's instructions.

#### 2.4.5. Histopathological assay

Ankle joint were fixed with 4% paraformaldehyde overnight, decalcified with 10% EDTA solution for 40 days. After dehydration, the samples were embedded in paraffin and sliced into 4 μm-thick sections. Tissue sections were prepared and stained with hematoxylin and eosin (H&E, Sigma Aldrich), and observed under light microscope.

#### 2.4.6. *In vivo* safety assessment

To evaluate the safety of each formulation, the serum levels of AST (aspartate aminotransferase), ALT (serum alanine aminotransferase), CRE (creatinine), and BUN (blood urea nitrogen) were measured by using the standard kits according to the manufacturer's instructions. At the same time, the livers, kidneys and testis were used for histological analysis as described above.

### 2.5. Statistical analysis

The data were represented as mean ± standard error of the mean (SEM). All data were analyzed by one-way analysis of variance (ANOVA). A value of *p* < .05 was considered statistically significant.

## 3. Result and discussion

### 3.1. Characterization of the nanoparticles

#### 3.1.1. Particle size, zeta potential, morphology, EE and DL

**Figure 1.**
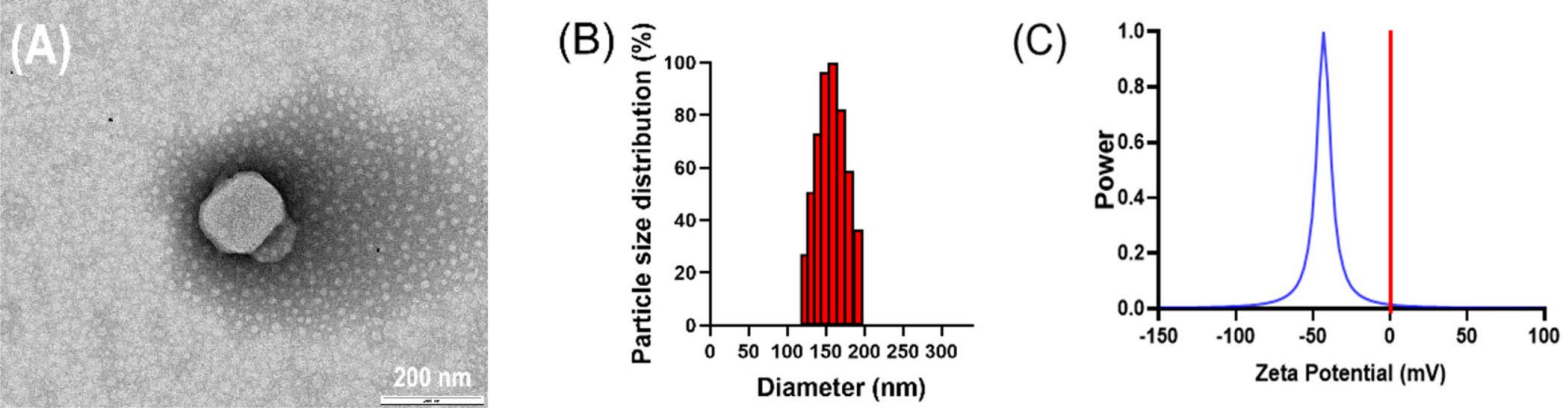
The TEM micrographs (A), particle size (B) and zeta potential (C) of the nanoparticles.

TP-VP NPs was prepared by using VP, cholesterol, and triptolide at an optimal weight ratio of 2:3:0.9. The morphology of TP-VP NPs was spherical and uniform (Figure 1A). The mean diameter of TP-VP NPs was 154 ± 2 nm with a PDI of 0.112 ± 0.026. The narrow particle-size distribution further validated the homogeneity of the nanoparticles (Figure 1B). The zeta potential was detected at −45.22 ± 0.92 mV (Figure 1C). Zeta potential is one of vital parameter to evaluate the nano-system stability, because the static electricity on the surface of nanoparticles could prevent the particles aggregation via electrostatic repulsion. In addition, the encapsulation efficiency of TP and VP in TP-VP NPs was 71.36 ± 2.73% and 93.45 ± 0.49%, the drug loading was 6.77 ± 0.99% and 26.37 ± 1.51%, respectively.

### 3.2. Stability of the nanoparticles

The particle size, PDI and zeta potential of TP-VP NPs were determined for 48 h incubation with PBS or DMEM with 10% FBS at 37 °C (A-C); and determined for 8 days in PBS at 4 or 25 °C (D-F).

**Figure 2.**
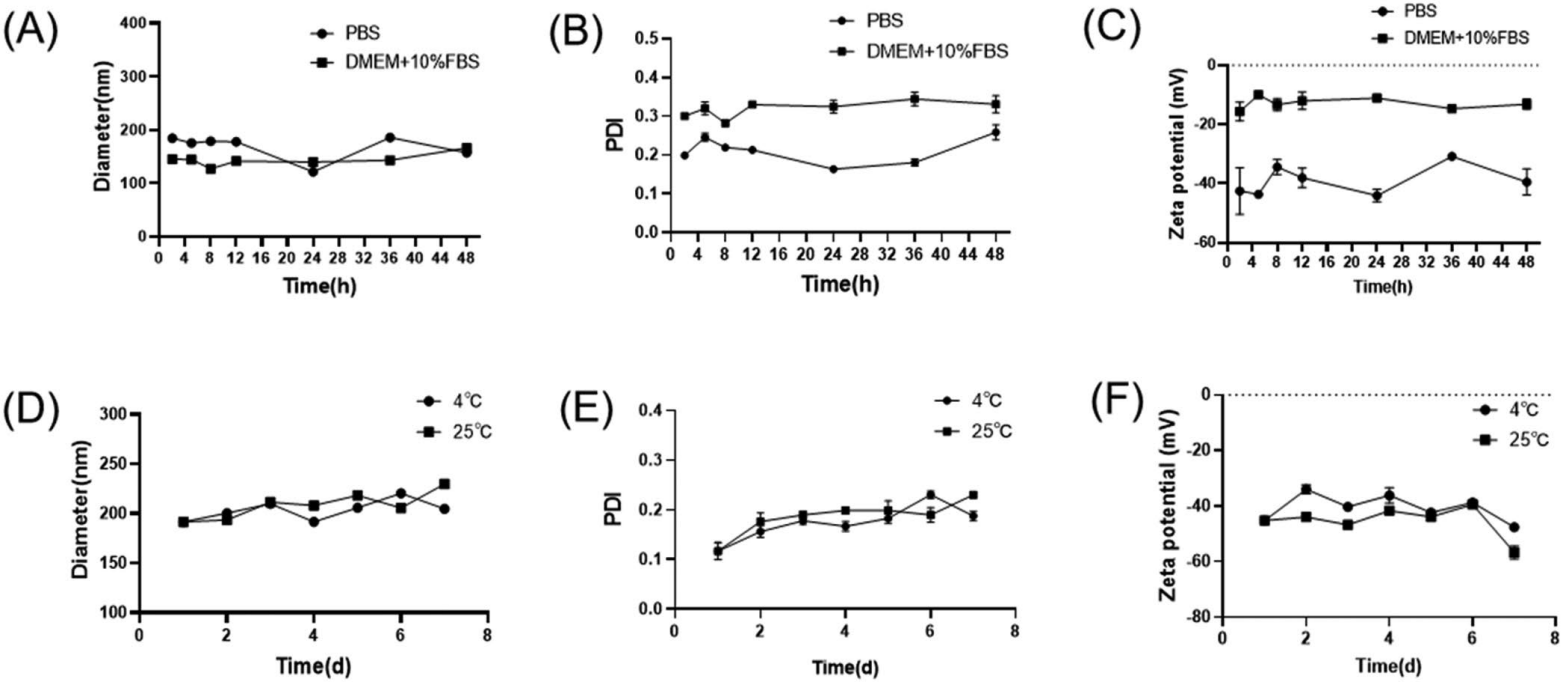
The physical stability of the nanoparticles in different storage conditions (*n* = 3).

The physical stability of TP-VP NPs was investigated in different storage conditions. Figure 2(A–C) showed that the particle size and PDI of the nanoparticles did not change significantly in PBS and DMEM medium containing 10% FBS for 48 h. The diameter was in the range of 145 ± 1 nm to 190 ± 1 nm between PBS and DMEM medium, and PDI was from 0.17 ± 0.01 to 0.33 ± 0.02. Meanwhile, the zeta potential was increased from about −45 mV to −15 mV, indicating that the zeta potential of TP-VP NPs was greatly affected by the different storage medium. However, the parameter values of the particle size, PDI and zeta potential in DMEM medium changed ever so slightly during 48 hours in comparison with PBS. The results suggested that the nanoparticles are still stabile in DMEM medium containing 10% FBS. When TP-VP NPs were incubated in PBS at 4 °C or 25 °C, there were little change in the particle sizes, PDI and zeta potential, indicating that the nanoparticles remained intact in FBS even during 7 days (Figure 2D–F).

### 3.3. *In vitro* release study

**Figure 3.**
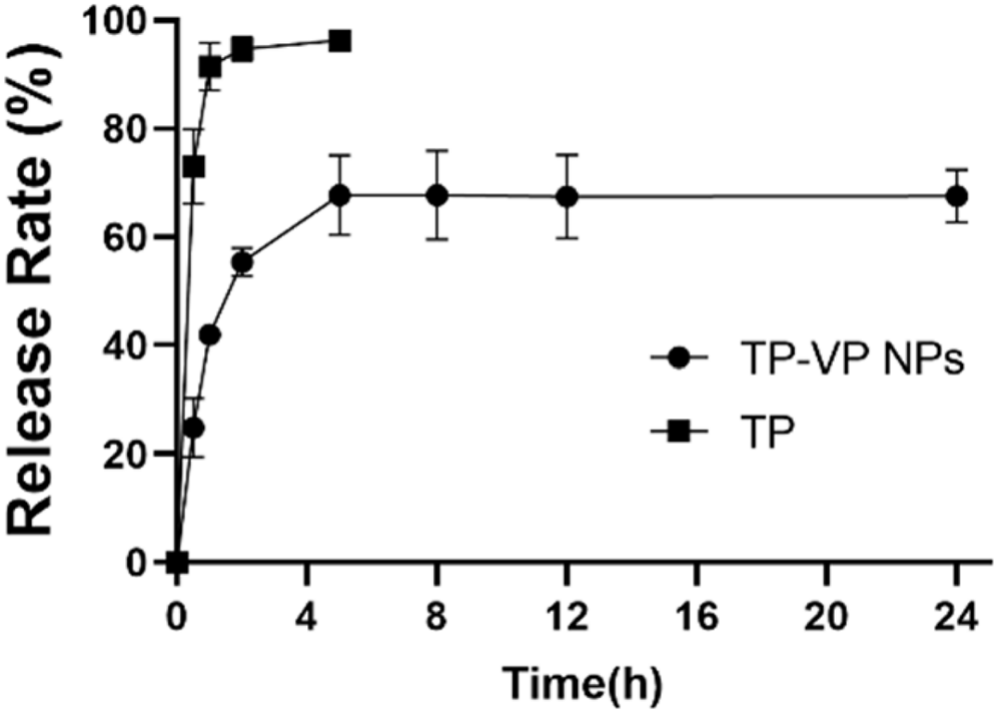
The cumulative release of TP from TP-VP NPs and TP solution in PBS at 37 °C.

The *in vitro* drug release profiles of TP-VP NPs and free TP illustrated that the free TP was exude from the dialysis bag in a burst-release manner within 2 hours, whereas TP-VP NPs undergone a sustainable manner (Figure 3). The release rate of free TP from the dialysis bag was over 95% within the initial 2 hours, followed by reaching a plateau (approximately 100%). Approximately 50% of TP was released from TP-VP NPs in the initial 2 h, and about 70% until 24 h, indicating a sustained drug release from nanoparticles. TP-VP NPs might maintain a suitable blood drug concentration in comparison with the free TP, which would greatly reduce the administration dosage and the usage frequency.

### 3.4. *In vivo* therapeutic efficacy on CIA mice

**Figure 4.**
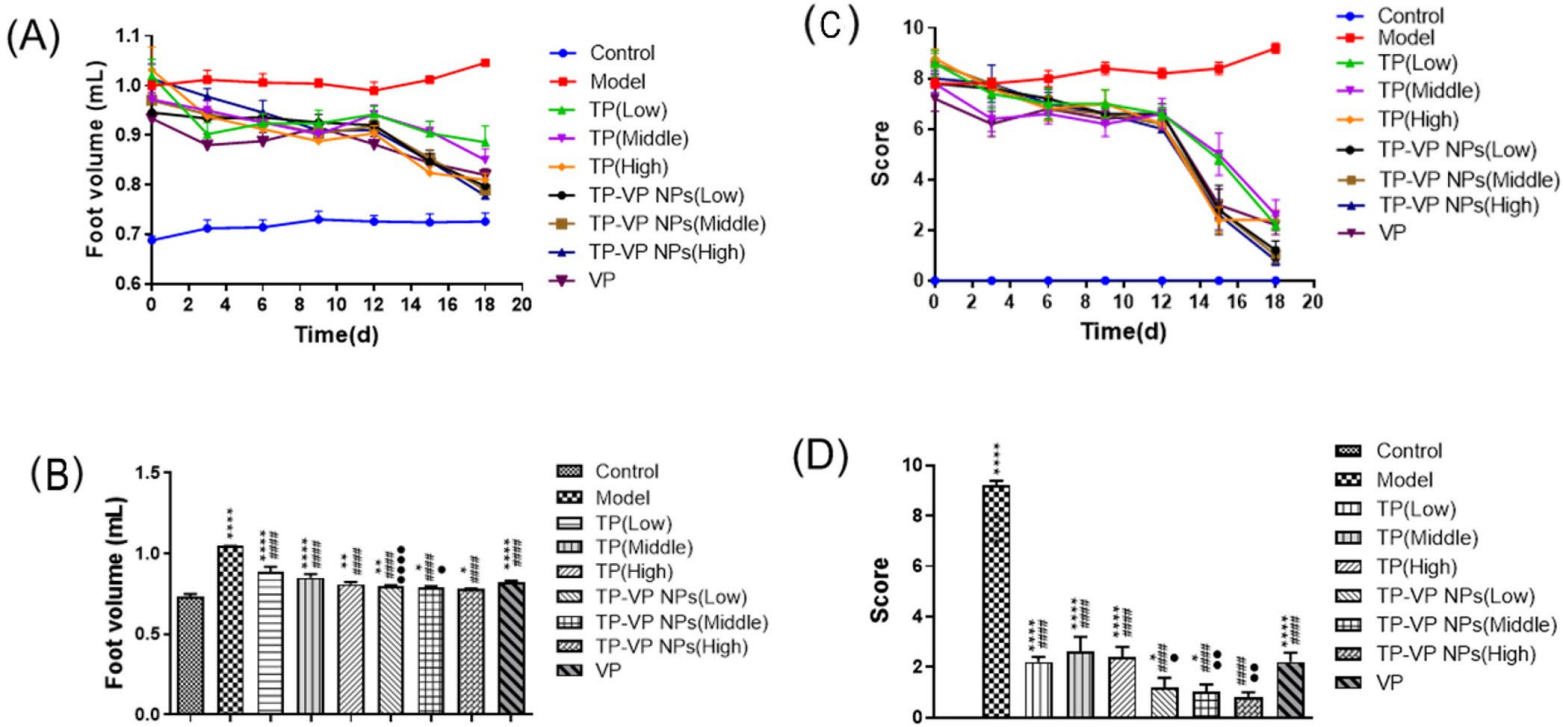
Changes of foot volume and clinical arthritis score during administration (A), (C) and on the last day (B), (D). * Represents *p* < .05,** represent *p* < .005, ^****^ represent *p* < .0001 vs control group; ^####^ represent *p* < .0001 vs model group; ^●^ represent *p* < .05, ^●●^ represent *p* < .005, ^●●●●^ represent *p* < .0001 vs TP group.

The arthritis symptoms were described by the foot volume and clinical arthritis scores, as shown in Figure 4. The foot volume of the treated arthritic mice had the similar size except the model group and the control group (Figure 4A). At the end of the experiment, the foot volume in the treatment groups was lower than that of the model group, and the TP-VP NPs group showed lower foot volume in comparison with the TP group (Figure 4B, *p* < .05). Those results indicated that TP-VP NPs might exert a better therapeutic effect via the synergistic effect of TP and VP. Moreover, the shape and joint swelling of the feet in CIA mice were evaluated by the arthritis index (Figure 4C and D). The treatment groups showed a significantly increasing detumescence rate of the feet, compared with the model group (Figure 4C). Furthermore, the reduction of the clinical arthritis score in the TP-VP NPs group was more notable than in other groups (*p* < .05) at the end of the experiment (Figure 4D). The results were greatly in agreement with those in the foot volume assay. The clinical arthritis score in the TP-VP NPs group reached closer to control group at the end of the experiment, which indicated that TP-VP NPs could more quickly alleviate the joint swelling of CIA mice than TP.

#### 3.4.1. Effect of TP-VP NPs on the inflammation factors

**Figure 5.**
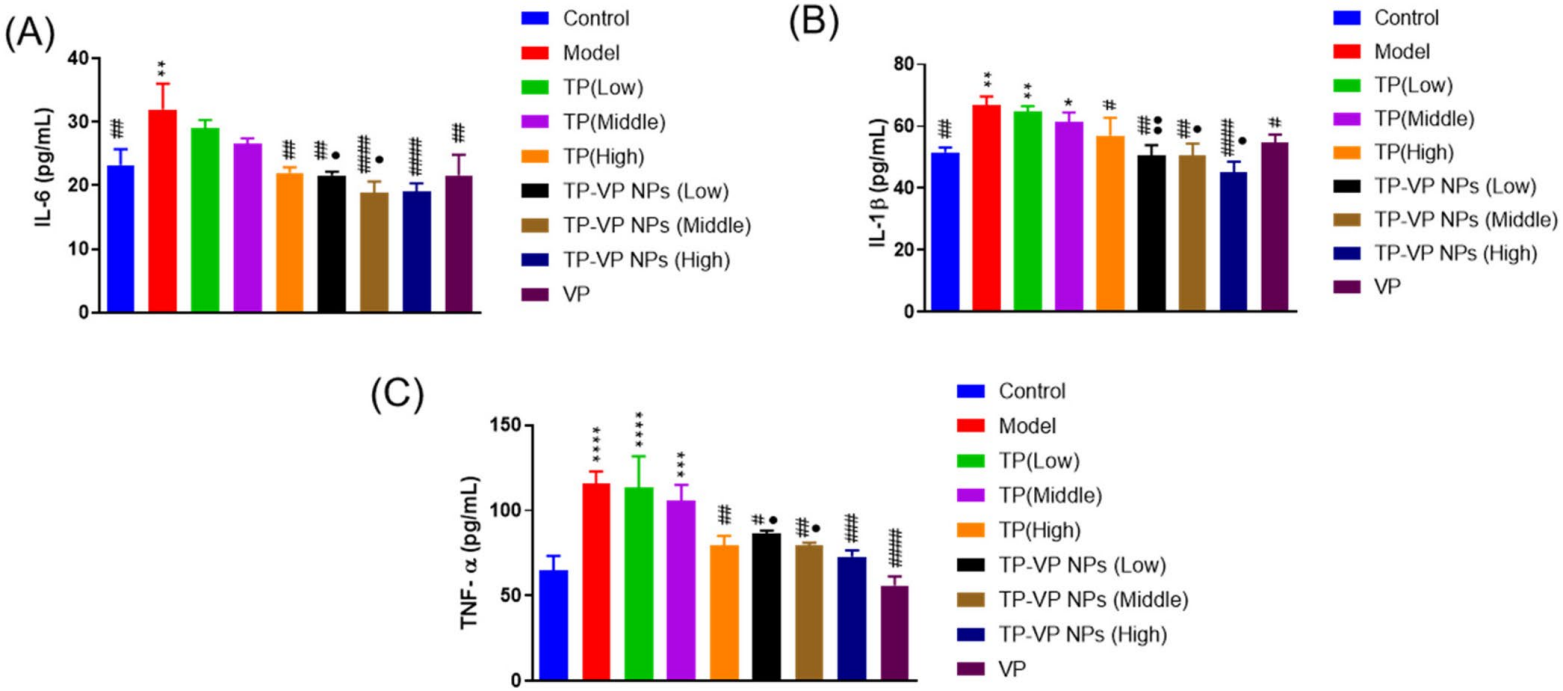
Effect of TP-VP NPs on the inflammation factors. * Represent *p* < .05; ** represent *p* < .005; *** represent *p* < .001; ^****^ represent *p* < .0001 vs control group; ^#^ represent *p* < .05; ^##^ represent *p* < .005; ^###^ represent *p* < .001; ^####^ represent *p* < .0001 vs model group; ^●^ represent *p* < .05; ^●●^ represent *p* < .005 vs TP group.

The inflammatory cytokines in inflamed joints are usually released into the blood. Hence, the serum levels of TNF-α, IL-6 and IL-1β were determined to verify the effect of TP-VP NPs on inhibiting the production of inflammatory cytokines (Figure 5). Compared with the model group, TP-VP NPs and VP groups showed significant reduction of the serum levels of IL-1β, IL-6 and TNF-α (*p* < .05), while the TP group exhibited only slight reduction of those inflammatory cytokines except the high-dose TP group (*p* > .05). The results showed that TP, VP and TP-VP NPs could reduce the IL-1β, IL-6 and TNF-α levels in the CIA-induced mice. Compared with TP and/or VP, TP-VP NPs showed the most significant reduction of inflammatory cytokines in a dose-dependent manner, suggesting that TP-VP NPs might exert anti-inflammatory effects through the synergistic effect of TP and VP. In this regard, further studies are warranted to confirm the synergistic effect of TP and VP against CIA-induced inflammatory.

#### 3.4.2. Effect of TP-VP NPs on oxidative stress in liver

**Figure 6.**
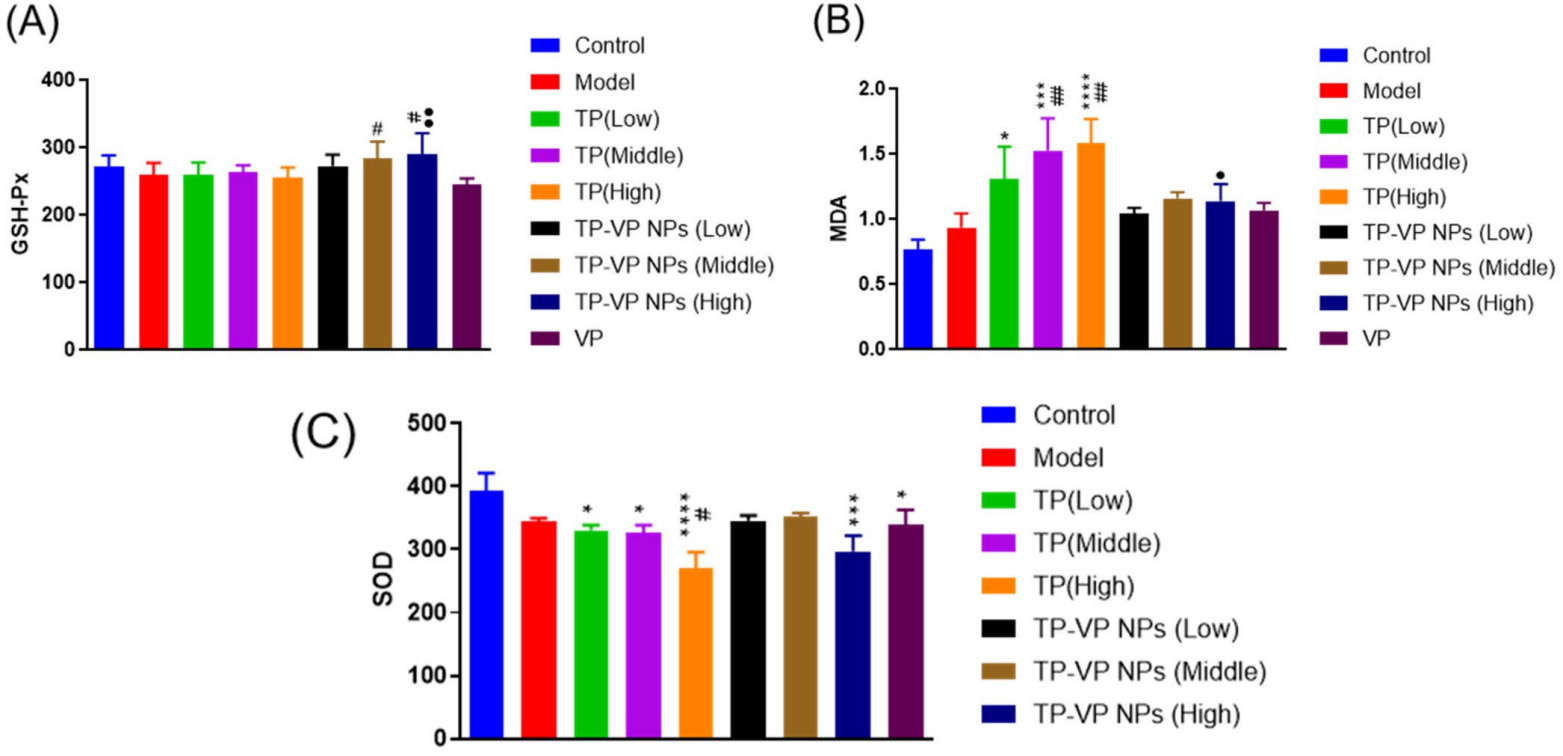
Effect of TP-VP NPs on oxidative stress. * Represent *p* < .05; *** represent *p* < .001; ^****^ represent *p* < .0001 vs control group; ^#^ represent *p* < .05; ^##^ represent *p* < .005 vs model group; ^●^ represent *p* < .05; ^●●^ represent *p* < .005 vs TP group.

To confirm the protective effects of TP-VP NPs on the oxidative stress, we examined the levels of the MDA, SOD and GSH-Px in the liver (Figure 6). The hypodermic injection of the bovine type II collagen emulsion induced the slight increase of the MDA level, and the slight reduction of the SOD and GSH-Px levels in the model group (*p* > .05). However, there were significant increase of the MDA level (*p* < .05, *p* < .001, *p* < .0001, vs the control group; *p* < .005, vs the model group) and significant decrease of the SOD level (*p* < .05, *p* < .0001, vs the control group; *p* < .05, vs the model group) after the CIA mice treated with TP. Compared with the TP group, the GSH-Px level significantly increased, while the MDA level decreased much more in the TP-VP NPs group (*p* < .05, *p* < .005, vs the TP group). However, there were not significant differences between the TP-VP NPs group and the TP groups in the SOD levels (*p* > .05). Vitamin C exhibits anti-inflammatory and antioxidant effects (Hasan Khudhair et al., 2022), while TP has mainly anti-inflammatory effect. In summary, the antioxidant enzyme levels in the CIA mice with TP-VP NPs treatment tended to return to normal, indicating that TP-VP NPs could ameliorate significantly the antioxidant stress in the liver.

#### 3.4.3. Effect of TP-VP NPs on joint histopathological changes

**Figure 7.**
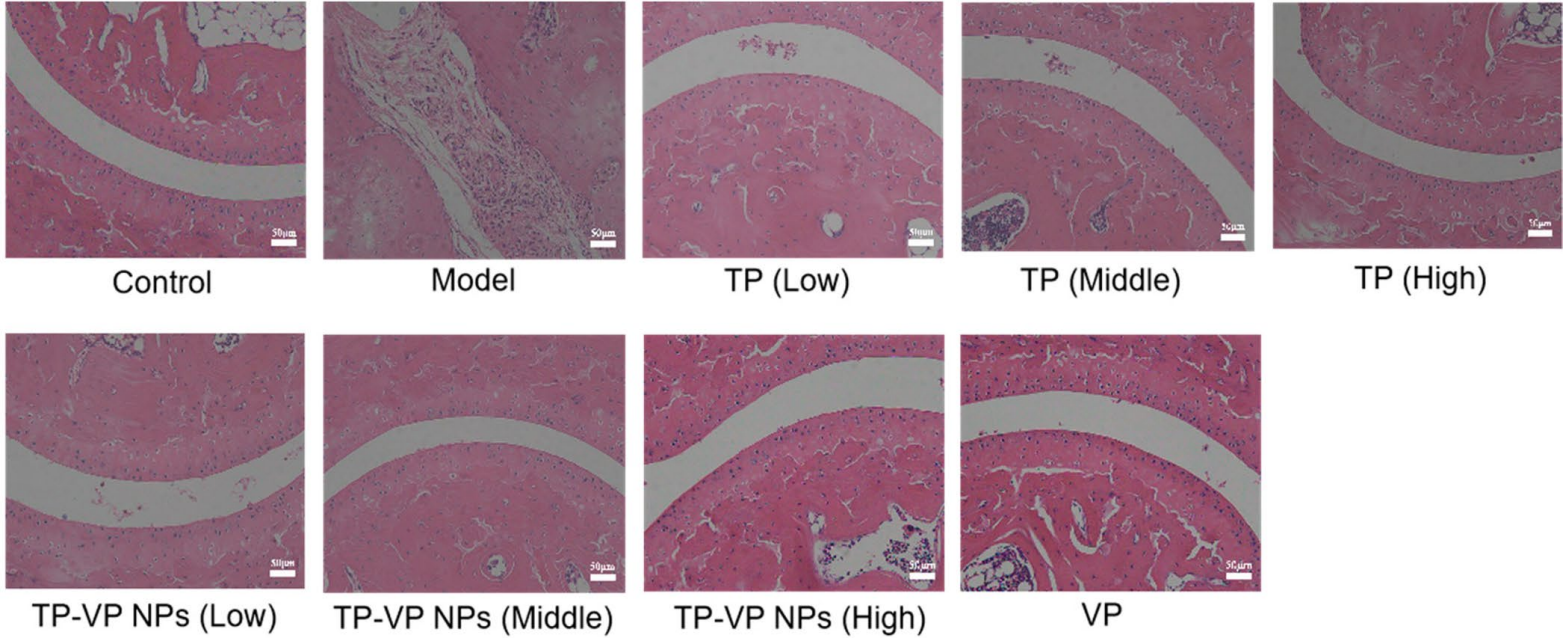
Effect of TP-VP NPs on joint histopathological changes.

Histopathological examination showed that the bone and cartilage tissues in the model group were seriously damaged, the boundary was ambiguous, and inflammatory cell infiltration was very obvious (Figure 7). However, the bone and synovium of the treated mice were relatively intact, and the inflammatory cell infiltrations were not observed in the mice treated with the middle- and high-dose TP-VP NPs and VP (Figure 7), indicating that VP and TP-VP NPs could alleviate effectively the pathology changes in inflamed joints by down-regulating inflammatory cytokine levels.

### 3.5. *In vivo* safety evaluation

**Figure 8.**
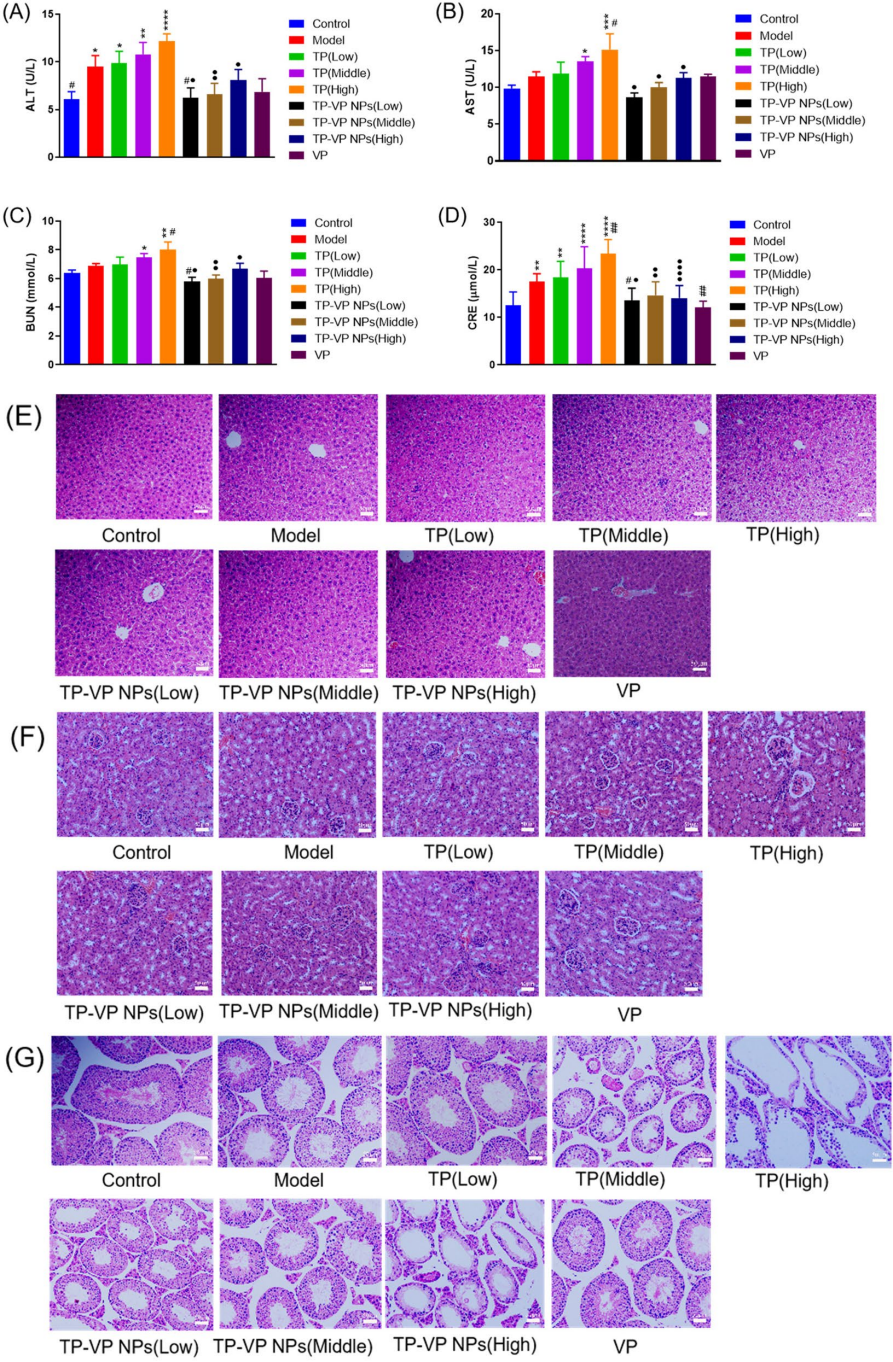
Safety evaluation *in vivo*. The level of AST (A), ALT (B), BUN (C), CRE (D); the H&E staining of liver tissue (E), renal tissue (F), testicular tissue (G). * Represent *p* < .05; ** represent *p* < .005; *** represent *p* < .001; ^****^ represent *p* < .0001 vs control group; ^#^ represent *p* < .05; ^##^ represent *p* < .005 vs model group; ^●^ represent *p* < .05; ^●●^ represent *p* < .005; ^●●●●^ represent *p* < .0001 vs TP group.

For the safety concerns, the hepatotoxicity was evaluated by testing the serum alanine transaminase (ALT) and aspartate aminotransferase (AST) (Figure 8A and B), and the nephrotoxicity was estimated by testing blood urea nitrogen (BUN) and creatinine (CRE) (Figure 8C and D). The results demonstrated that the AST, ALT, BUN, and CRE levels in the TP groups were significantly higher than those in the control group and other treatment groups (*p* < .05), indicating that TP significantly induced liver and kidney injury in a dose-dependent manner. However, there were no obvious differences between the TP-VP NPs groups, VP group and the control group (*p* > .05), which indicated that VP was harmless or low toxic as a drug carrier, and TP-VP NPs could significantly reduce the hepatorenal toxicity induced by TP in CIA mice.

Histopathological analysis explored whether the drugs led to any adverse effects on the liver, kidney and testicular tissues of the ICA mice (Figure 8E–G). Compared with the control group, some histopathological changes were observed in the major organs of the model mice, which indicated that the bovine type II collagen had sensitization and slight systemic toxicity. In the TP groups, the hepatocytes arranged in disorder, accompanied with nuclear pyknosis, loose cytoplasm, and inflammatory cell infiltration in a dose-dependent manner (Figure 8E). Compared with the TP groups, the liver lesions of mice were significantly alleviated in the low- and medium-dose TP-VP NPs groups, which indicated that TP-VP NPs could decrease the liver injury induced by TP.

The kidney pathology analysis showed the atrophy of the glomeruli, the collapse of basement membrane and even the occlusion of lumen in the TP groups (Figure 8F). In contrast, the renal lesions were significantly reduced by TP-VP NPs in a dose-dependent manner, which indicated that TP-VP NPs could reduce the renal injury induced by TP. The testicular histopathology depicted that the seminiferous tubules were atrophied and twisted into irregular shape in the TP groups, and the spermatogenic cells as well as sperm cells in the seminiferous tubules were significantly less than those in the control group (Figure 8G). Furthermore, the pathological injury of testis was gradually aggravated in a dose-dependent manner. Compared with TP groups, the pathological changes of testis were significantly reduced in the low- and medium-dose TP-VP NPs groups, which was also consistent with the liver and kidney histopathological analysis as mentioned above. In addition, the VP groups displayed no significant histopathological changes in the major organs, compared with the control group, indicating that VP play an important role in the reduction of the systemic toxicity induced by the bovine type II collagen as well as TP.

## 4. Conclusions

In summary, we successfully developed TP-VP NPs that could alleviate the joint inflammation induced by collagen and reduce significantly the systemic toxicity induced by triptolide for effective RA treatment. TP-VP NPs have good physical stability and sustained drug-release effect. TP-VP NPs could down-regulate IL-1β, IL-6 and TNF-α levels to inhibit the erosion of synovitis and bone tissue, and alleviate the swelling and deformation of CIA mice’s feet, and ameliorate significantly the antioxidant stress in the main organs. TP-VP NPs could effectively alleviate the toxicity injury of TP, which did not cause much damage to the liver and kidney, compared with the TP. Our results demonstrated that TP-VP NPs is a promising triptolide delivery system for the treatment of RA, which enhances the solubility of free TP and reduces the toxicity of TP *in vivo*.
